# Characterization of RNA-Like Oligomers from Lipid-Assisted Nonenzymatic Synthesis: Implications for Origin of Informational Molecules on Early Earth

**DOI:** 10.3390/life5010065

**Published:** 2015-01-05

**Authors:** Chaitanya V. Mungi, Sudha Rajamani

**Affiliations:** Indian Institute of Science Education and Research (IISER), Pune, Maharashtra 411008, India; E-Mail: cvmungi@students.iiserpune.ac.in

**Keywords:** dehydration-rehydration cycles, prebiotic chemistry, lipid-assisted polymerization, abasic sites, sugar-phosphate backbones

## Abstract

Prebiotic polymerization had to be a nonenzymatic, chemically driven process. These processes would have been particularly favored in scenarios which push reaction regimes far from equilibrium. Dehydration-rehydration (DH-RH) cycles are one such regime thought to have been prevalent on prebiotic Earth in niches like volcanic geothermal pools. The present study defines the optimum DH-RH reaction conditions for lipid-assisted polymerization of nucleotides. The resultant products were characterized to understand their chemical makeup. Primarily, our study demonstrates that the resultant RNA-like oligomers have abasic sites, which means these oligomers lack information-carrying capability because of losing most of their bases during the reaction process. This results from low pH and high temperature conditions, which, importantly, also allows the formation of sugar-phosphate oligomers when ribose 5'-monophosphates are used as the starting monomers instead. Formation of such oligomers would have permitted sampling of a large variety of bases on a preformed polymer backbone, resulting in “prebiotic phosphodiester polymers” prior to the emergence of modern RNA-like molecules. This suggests that primitive genetic polymers could have utilized bases that conferred greater N-glycosyl bond stability, a feature crucial for information propagation in low pH and high temperature regimes of early Earth.

## 1. Introduction

Informational molecules like nucleic acids play a crucial role in modern biology. Fundamental functions in cells like catalysis, information storage, and regulation of various processes are carried out by these specialized polymers. Formation of these polymers in itself is a very complex process that is orchestrated by enzymes, which use triphosphate-activated moieties as the starting monomers. Prebiotic polymerization, on the other hand, had to be a nonenzymatic, chemically driven process facilitated by a series of condensation reactions. One such set of reactions would have resulted in phosphodiester bonds similar to those that are encountered in today’s informational polymers. Since the condensation reaction involves the loss of a water molecule, it is highly unfavorable in aqueous solutions. Reverse hydrolysis of the resultant bonds occurs at a greater rate in such scenarios, limiting the polymerization potential of the monomers in question. Therefore, mechanisms that push reaction regimes far from the equilibrium are pertinent as they sequester newly formed polymers in a kinetic trap, where the rate of synthesis exceeds the rate of hydrolysis. Previous studies undertaken in prebiotic chemistry have used imidazole-activated nucleotides to investigate nonenzymatic polymerization reactions [1]. This type of activation chemistry allows for faster polymerization, resulting in higher yields of oligomers. Various catalytic environments such as clay (montmorillonite) [2] and eutectic ice phases [3] have been shown to promote polymerization of these activated nucleotides. However, the presence of imidazole-activated nucleotides in high concentrations on prebiotic Earth is debatable. On the contrary, formation of nucleotides has been demonstrated in prebiotically relevant schemes [4,5]. Therefore, characterizing conditions that favor polymerization of non-activated nucleotides is of great interest as it could enable us to delineate schemes that might have led to the origin of informational polymers.

Fluctuating environments are thought to have been a common theme on prebiotic Earth. For example, recurrent geological activity would have resulted in large fluctuations in the environment, resulting in landscape and parameter changes that defined a specific reaction regime. In particular, volcanic activity could have resulted in cyclic changes in the environment, facilitating interesting reaction possibilities [6]. Dehydration-rehydration (DH-RH) cycles would have been one such regime that might have driven uphill reactions like polymerization [7]. DH-RH cycles could have resulted from temperature fluctuations due to day-night cycles, seasonal variations, or by action of waves at the edge of oceans. Volcanic geothermal pools and inter-tidal pools are important prebiotic environmental niches where DH-RH cycles are thought to have allowed for interesting chemistry to occur. Dehydration acts as a concentrating mechanism which promotes condensation reactions and the loss of water in such scenarios is promoted by higher temperatures. This reduces the chance of back hydrolysis, resulting in the accumulation of polymers over time. Few previous studies have attempted to polymerize amino acids under dehydrated conditions [8]. Poor yields obtained in these studies can be attributed to the difficulty of forming amide bonds under these conditions [9]. Alpha hydroxy acids such as glyceric acid and lactic acid have also been shown to polymerize under dehydrated conditions [10,11]. More recently, the formation of oligoesters was demonstrated when malic acid was subjected to DH-RH cycles at a moderately high temperature [12]. The aforementioned studies support the idea that DH-RH cycles can facilitate a kinetic trap that results in the formation of oligomers.

In one especially relevant study, nucleoside 5'-monophosphates (5'-NMPs) were shown to polymerize when subjected to DH-RH cycles in the presence of phospholipids [13]. When analyzed using a nanpore detector, which is used for single molecule RNA analysis, the resulting polymers showed characteristic blockades similar to those of a control anionic polymer (PolyA 50mer). In addition, denaturing polyacrylamide gel electrophoresis (PAGE) was used to analyze the resultant products after radiolabeling with ^32^P. This indicated the RNA-like nature of these oligomers (seen as streaks on the gel), which corresponded in length to an RNA of 50–75 nucleotides. The streaking was attributed to the complexity of the products formed, which hindered resolution of the oligomers into specific bands. Lipids were hypothesized to organize the reacting monomers in two-dimensional arrays where their orderly arrangement favors polymerization. This was supported by an X-ray diffraction study that confirmed that the 5'-AMP molecules are indeed organized between bilayers, with reactive groups present in close proximity [14]. In addition, templated information transfer was also found to be plausible using lipid-assisted DH-RH cycles of non-activated nucleotides [15]. In a very recent paper, the same group demonstrated that ds-RNA is relatively resistant to hydrolysis when subjected to DH-RH cycles at high temperatures [16]. This study also reported the formation of oligomers capable of base pairing when cognate Watson–Crick monomers were used as the starting reactants. This was indicated by hyperchromicity experiments and their detection on denaturing gels using intercalating dyes. The mechanism underlying these polymerization reactions is thought to be similar to acid-catalyzed esterification, as in the case of carboxylic acids [15,16]. Variability observed in the nanopore and gel analyses suggested that the products formed in these reactions are a complex mixture of oligomers (e.g., both 2'-5'- and 3'-5'-linked RNA). However, the exact chemical nature of these RNA-like oligomer products is not known. In the present study, we characterize in detail the effect of various parameters on phospholipid-assisted polymerization of 5'-NMPs. We have explored a range of parameters that increase the efficiency of these reactions and have narrowed down the conditions under which polymerization is optimized. The resultant products were characterized using several biochemical techniques. Importantly, our study sheds light on the actual chemical nature of the resultant RNA-like oligomers and has significant implications for the role these molecules might have played in the origin of informational molecules in the RNA-like world.

## 2. Experimental Section

### 2.1. Materials

Adenosine 5'-monophosphate (AMP), uridine 5'-monophosphate (UMP), guanosine 5'-monophosphate (GMP), cytidine 5'-monophosphate (CMP), and ribose 5'-monophosphate (rMP) were purchased as disodium salts from Sigma-Aldrich (Bangalore, India) and used without further purification. Phospholipids including 1-palmitoyl-2-oleoyl-sn-glycero-3-phosphocholine (POPC) and 1,2-dilauroyl-sn-glycero-3-phosphocholine (DLPC) were purchased from Avanti Polar Lipids Inc (Alabaster, AL, USA). All other reagents used were of analytical grade and purchased from Sigma-Aldrich (Bangalore, India).

### 2.2. Methods

#### 2.2.1. Simulating Prebiotic Conditions

Early Earth conditions were simulated by assembling a bench-top heating block that was enabled to maintain high temperatures and anaerobic environment. The heating block was fitted with adaptors to accommodate several vials that contained the reaction mixtures. Reaction vials were secured with caps that had PTFE septa in them purchased from Chemglass (Vineland, NJ, USA), through which PEEK tubing of about 1–1.5 inches was inserted to deliver CO_2_ into the vials. A second similar PEEK tube, which was also inserted into the septum, served as an escape vent for initial air and the excess CO_2_ and water vapors generated during the reaction. The heating block was maintained at a specified temperature and the reaction mixtures were dried under CO_2_. After the dehydration phase was over, rehydration was performed by introducing rehydrating agents through a third PEEK tube. Multiple DH-RH cycles were performed to study the effect of different parameters on lipid-assisted polymerization reaction of ribonucleotides, and reaction mixtures were analyzed after several cycles using the biochemical techniques below. All experiments were performed at least twice to confirm the trends that were observed in the reactions.

#### 2.2.2. HPLC Analysis

HPLC analysis was performed on the aqueous phase of the reaction mixtures that were obtained post butanol-hexane extraction to remove phospholipids [13]. Chromatography was performed using an Agilent 1260 chromatography system (Agilent Technologies, Santa Clara, CA, USA) and DNAPac PA200 column (4 mm × 250 mm) from Dionex (Thermo Scientific, Sunnyvale, CA, USA). Samples were analyzed with a linear gradient of NaClO_4_ in 2 mM Tris buffer at pH 8 using a flow rate of 1 mL/min. All solvents, purchased from Sigma-Aldrich (Bangalore, India), were of HPLC grade and used after filtering through a 0.22-µm nylon filter followed by degassing. Samples were detected using a high-sensitivity flow cell (60 mm path length) in a diode array detector. In the case of some samples, a fluorescence detector was used for analysis.

#### 2.2.3. Mass Analysis

Mass analysis was performed using Matrix-Assisted Laser Desorption Ionization (MALDI) and high-resolution mass spectrometry (HRMS). MALDI was performed on aqueous phase samples using 2′,4′,6′-Trihydroxyacetophenone (THAP) as matrix on 4800 Plus MALDI TOF/TOF™ Analyzer (AB SCIEX, Framingham, MA, USA). Attempts to reduce salt from reaction mixtures were made by using C_18_ Zip-Tips (Merck KGaA, Darmstadt, Germany). HRMS analysis was carried out using the Acquity UPLC^+^ system from Waters, with an Alltima C18 column (2 µm, 2.1 mm × 50 mm) with a water/acetonitrile gradient containing 0.1% formic acid. Mass determination was carried out in the positive ion mode with SYNAPT G2 Mass Spectrometry (Waters, Milford, CT, USA).

## 3. Results

### 3.1. HPLC Analysis of Products

**Figure 1:**
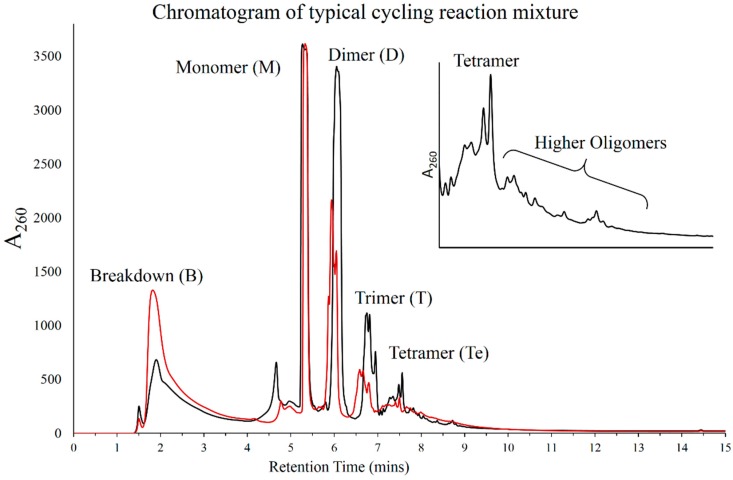
HPLC chromatogram of Cyc7 sample from a typical DH-RH reaction containing only 5 mM of 5'-AMP (red trace) or 5 mM of 5'-AMP + 1 mM POPC (black trace). The identity of the peaks has been indicated. Inset shows tetramer cluster followed by a trail of unresolved higher oligomers. Trailing could result from low yields and high variability within each oligomer of a specific length.

Previous reports have suggested that HPLC analysis of these products is difficult due to variability in the chemical nature of the products formed, which, in turn, has a bearing on the resultant yields of the individual oligomers. We, however, carried out analysis of these reaction mixtures using DNAPac PA200 column. It is an anion exchange column that offers single nucleotide resolution and has been used extensively to analyze oligomers resulting from polymerization of activated nucleotides [17,18]. The gradient was standardized using 5'-AMP as a monomer control and hydrolyzed PolyA to ensure that the salt gradient was optimized for resolving smaller oligomers. A typical reaction mixture was used for the standardization of all reaction parameters, which consisted of 5 mM 5'-AMP and 1 mM POPC. This was subjected to seven DH-RH cycles under acidic conditions using H_2_SO_4_ and the samples were analyzed by HPLC. Figure 1 shows the chromatogram for the resultant mixture obtained from this reaction. Multiple peaks were observed in the reaction mixture, some of which were identified based on their retention times with respect to the controls.

The first peak, which eluted in the dead volume without even interacting with the column, was termed as “breakdown” as this potentially could be adenine or adenosine, which would result due to thermal breakdown of 5'-AMP during the reaction. Subsequent peaks that eluted in the gradient were that of 5'-AMP monomer and higher oligomers of AMP, as indicated in Figure 1 (corroborated by mass analysis; details in Section 3.6). In most of the runs, peaks from dimer and above showed clustering, a feature that has been observed in products obtained from nonenzymatic synthesis of nucleotides [17]. This indicates the presence of multiple species in each of these oligomers, which possibly differ in their backbone linkages (2'-5' or 3'-5'). Also, the resolution was lost post tetramer peak cluster, beyond which only trailing was observed (as shown in figure inset). This potentially is a combination of low product yields and high variability within oligomers formed of a specific length. Given the following concerns including presence of a large breakdown peak, cluster formation in oligomers, and trailing post tetramers, we refrained from quantifying the yields of the reaction. Addition of lipids reduced the “breakdown” peak considerably and promoted polymerization (addressed in detail in Section 3.5). Since detection of oligomers was actually possible using HPLC, we decided to vary parameters that could affect lipid-assisted polymerization reactions to find an optimum regime where higher oligomer yields could be obtained.

### 3.2. Effect of pH and Rehydrating Agent on Polymerization

**Figure 2:**
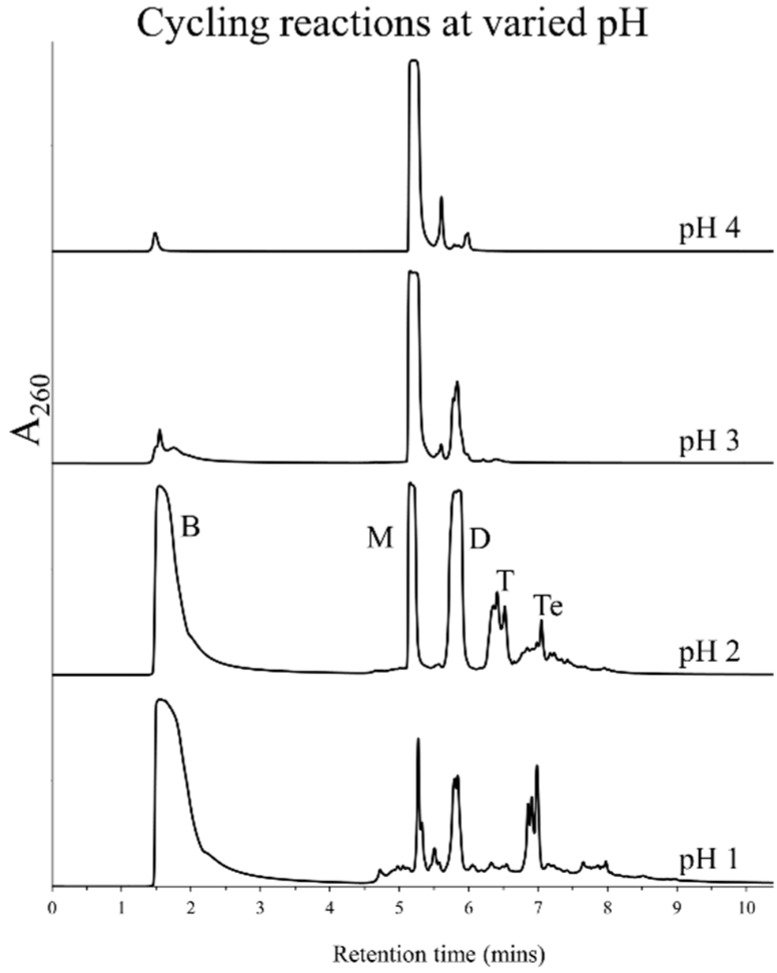
HPLC chromatograms obtained after seven DH-RH cycles using 5'-AMP (5 mM) and DLPC (1 mM) at specified pH values that were initially adjusted with H_2_SO_4_. Subsequent rehydrations were carried out with H_2_O. Polymerization is more efficient in reactions performed at a pH of 2 or below.

In order to optimize the reaction for obtaining better oligomer yields, several parameters were varied and their effects on the typical reaction mixture were studied. Since the mechanism proposed for this type of polymerization is similar to that of acid-catalyzed ester formation, we investigated the optimum pH conditions for polymerization of non-activated nucleotides. Mixtures of 5'-AMP, with and without lipids, were subjected to cycling at various pH values in the range of 1–4 (at intervals of 1 pH unit). The pH was adjusted to a particular value using H_2_SO_4_ and subsequent rehydrations were carried out using water. Polymerization of 5'-AMP was assessed using HPLC analysis. Figure 2 shows chromatograms that were obtained for cycling of 5'-AMP with DLPC at specified pH values. As seen in the figure, pH values above 2 did not produce good oligomer yields. Polymerization was more efficient at a pH of 2 or below, resulting in oligomers which elute from the column along the salt gradient. A pH of 1, however, resulted in higher breakdown than pH 2, as was evident from comparative analysis of these HPLC chromatograms at 280 nm (data not shown). Suboptimal absorbance at this wavelength allowed for better comparison of the breakdown peaks, which did not saturate, unlike what was seen at 260 nm (Figure 2, last two chromatograms). These results indicated pH 2 to be the optimum pH for polymerization of 5'-AMP under these conditions. At this pH, the phosphate group in 5'-AMP is thought to be protonated, enabling nucleophilic attack by a neighboring nucleotide’s 2'-OH or 3'-OH, resulting in a phosphodiester bond [15,16].

**Figure 3:**
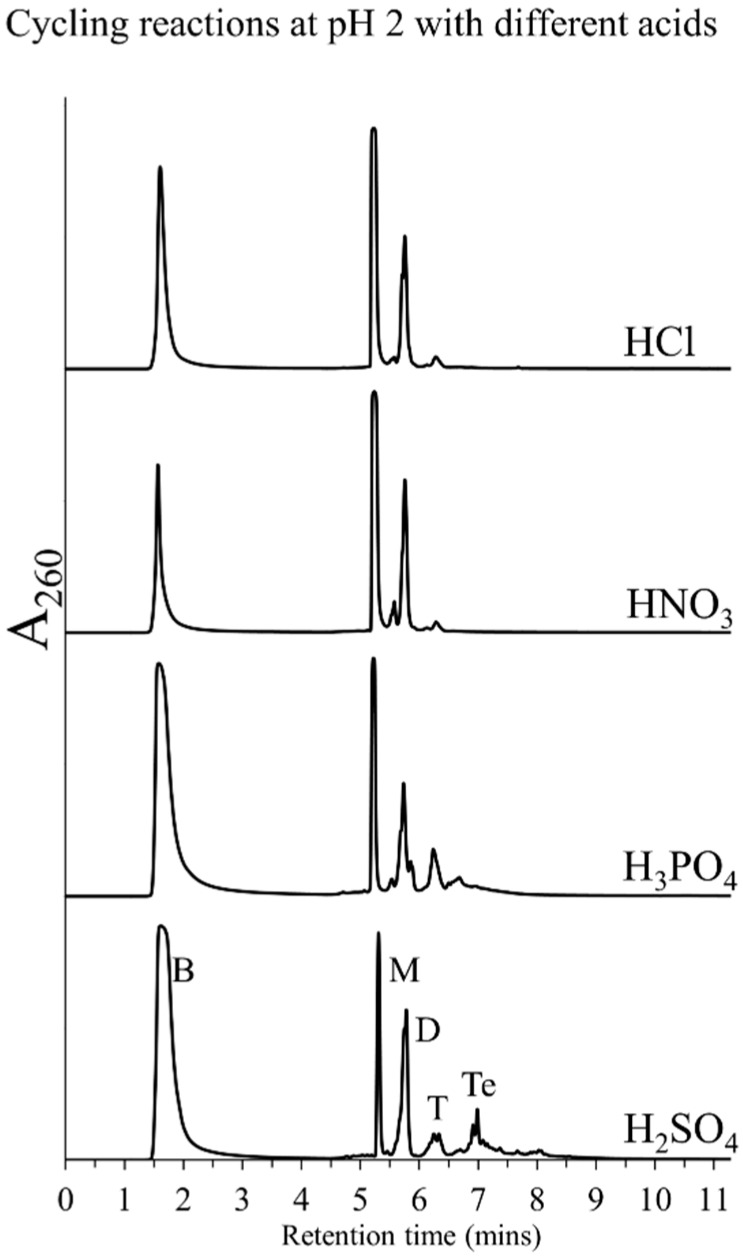
HPLC chromatograms obtained from Cyc7 samples of DH-RH reactions of 5'-AMP (5 mM) and POPC (1 mM) performed with various acids including HCl, H_2_SO_4_, HNO_3_, and H_3_PO_4_, at pH 2. Polymerization using H_2_SO_4_ and H_3_PO_4_ resulted in oligomers, while not much was observed with HCl and HNO_3_.

Since pH 2 was observed to be the optimum pH for polymerization, we next investigated if the acid used played any mechanistic role in the polymerization process. In other words, is polymerization favored by any acid that could bring the pH down to 2, or is it specific to only certain type of acids? To address this question, we carried out cycling with various mineral acids including hydrochloric acid (HCl), sulfuric acid (H_2_SO_4_), nitric acid (HNO_3_), orthophosphoric acid (H_3_PO_4_), and organic acids like formic acid and acetic acid. Some of these acids, e.g., HNO_3_ and formic acid, are known to be thermolabile and hence were replenished after every dehydration cycle in order to maintain pH ~2. As indicated in Figure 3, lesser polymerization was observed with HCl and HNO_3_, whereas H_2_SO_4_ and H_3_PO_4_ both resulted in reasonable oligomer yields. However, the two organic acids failed to promote any polymerization despite repeated addition of these acids over the cycling period.

The ability of various acids to promote polymerization seems to depend on the nature of the acid. Thermolabile acids or acids with low boiling point (HNO_3_, HCl, and the aforementioned organic acids) could not maintain low pH throughout the reaction, resulting in inefficient polymerization. H_2_SO_4_ is known to be a good dehydrating agent, which was the most potent in promoting polymerization, followed by H_3_PO_4_. For characterization of other parameters, in subsequent reactions, a pH of 2 was maintained using H_2_SO_4_ and rehydrations over DH-RH cycles were carried out using water.

### 3.3. Effect of Temperature on Polymerization

Temperature plays a crucial role in the rate of evaporation of water from a reaction. At lower temperatures, water will be lost slowly, resulting in increased water activity over the time course of the reaction. This will promote hydrolysis of newly formed oligomers, thus reducing oligomer yields. Higher temperatures, therefore, in principle, should result in higher yields. It should, however, be noted that the starting material itself may undergo thermal decomposition at very high temperatures (e.g., charring of sugars). This suggests that there might be an optimum temperature regime where forward reactions exceed unfavorable reactions, resulting in better oligomer yields. To test this, cycling was carried out at varying temperatures including 60 °C, 75 °C, 90 °C and 105 °C. The resultant mixtures from different cycles of these reactions were analyzed using HPLC (Figure S1, supplementary data). For simplicity, the HPLC chromatograms are represented graphically by using the area under relevant peaks as a proxy for comparing the oligomeric abundance observed in the different reactions. It is important to note that this area analysis does not reflect true oligomer yields due to technical concerns discussed in Section 3.1.

**Figure 4:**
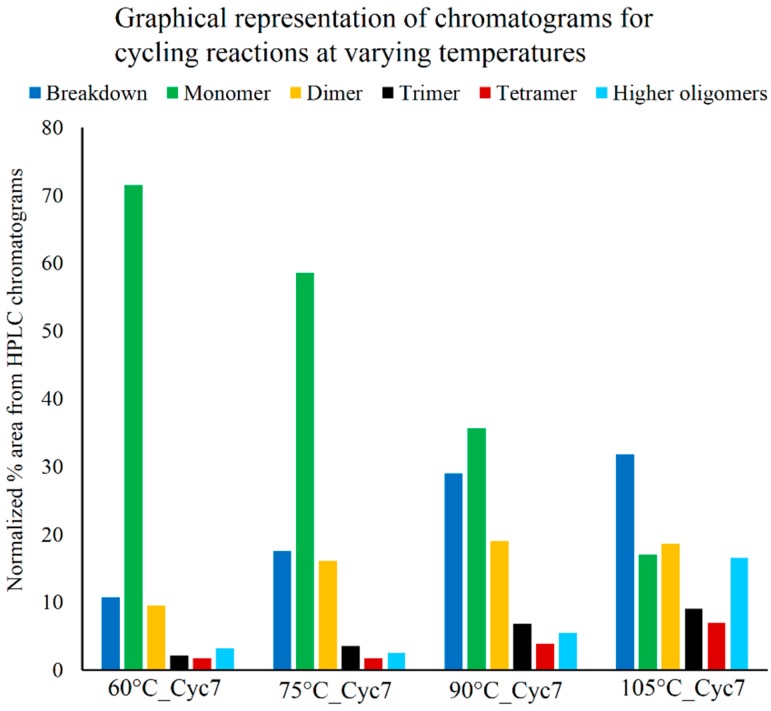
Graphical representation of HPLC chromatograms obtained from Cyc7 samples of DH-RH reactions of 5'-AMP (5 mM) and POPC (1 mM) performed at various temperatures (as indicated) at pH 2. The areas under relevant HPLC peaks are used as a proxy for comparing peak abundance observed in the different reactions. Higher temperatures (90 and 105 °C) promoted better polymerization but were also accompanied by greater breakdown.

At 60 °C, less polymerization was observed, which increased over cycles; a similar trend was also observed for the 75 °C reaction. In both cases, breakdown was less but so was the extent of polymerization. The cycling reaction that was carried out at 90 °C resulted in greater yields of oligomers even at lower cycles, which further improved with an increasing number of DH-RH cycles. However, at an even higher temperature of 105 °C, although polymerization was observed, oligomer yields did not alter much with subsequent cycles and the breakdown observed was also considerably high. On comparing reactions that were carried out for seven cycles at various temperatures (Figure 4), it was observed that higher temperatures promoted better polymerization, but this was also accompanied by greater breakdown (as seen in 105 °C reaction). Given our observations, it seems that temperatures of 80–90 °C are optimal for carrying out multiple DH-RH cycles.

### 3.4. Optimum Dehydration Time for Polymerization

Condensation events, which drive oligomerization, likely take place during the dehydration phase of the DH-RH cycles, while hydrolysis is promoted in the rehydration phase. Though a longer drying time can facilitate efficient polymerization, there also remains the possibility of thermal breakdown of the starting reactants over prolonged dehydration. On the other hand, too-frequent rehydration will reduce the oligomer yields by promoting hydrolysis. However, these rehydration phases are also necessary as they allow the formation of vesicles from dried lipid lamellae, which enable encapsulation of newly formed oligomers inside these vesicles. An interesting consequence is that this encapsulation could protect the growing oligomers from getting hydrolyzed, thereby potentially increasing yields of higher oligomers over several cycles. In addition, monomers become concentrated in the hydrophilic regions, close to the lipid head group where polymerization is promoted. In dehydrated conditions, these regions are surrounded by hydrophobic zones, formed by fatty acyl chains of the lipids, where monomers do not aggregate to polymerize. Rehydration phases are hence important to allow reactant molecules and the various growing oligomers to get shuffled around for facilitating increased polymerization over several DH-RH cycles. Consequently, there has to be an optimum regime of DH-RH that will balance these processes and favor forward reactions, resulting in greater oligomer formation.

**Figure 5:**
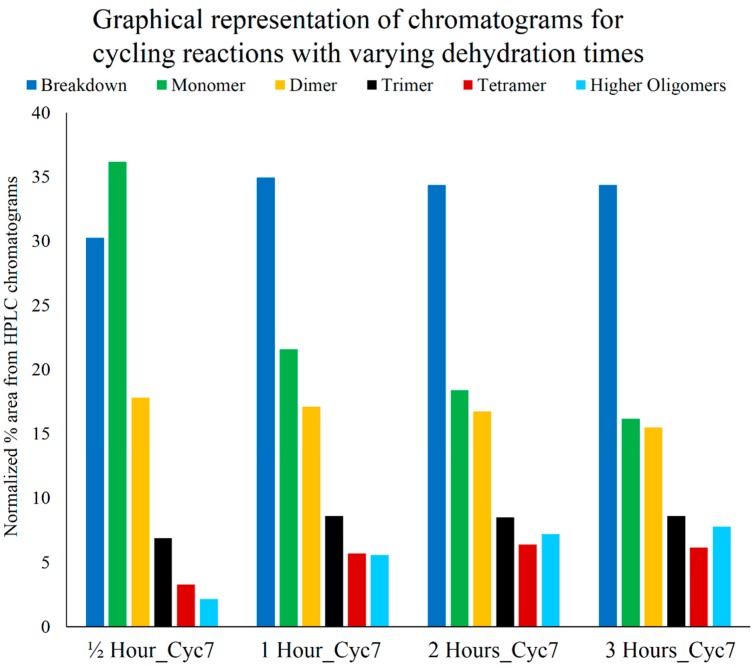
Graphical representation of HPLC chromatograms obtained from Cyc7 samples of DH-RH reactions of 5'-AMP (5 mM) and POPC (1 mM) carried out using varying dehydration time scales at pH 2. The optimum drying time for these reactions seems to be around 1 h.

To identify this, various dehydration time scales from 30 min to 3 h were analyzed. It was observed that longer dehydration time periods did indeed result in higher yields of oligomers, as shown in Figure 5. However, increasing the dehydration time period to over 2 h resulted only in a marginal increase in yields of higher oligomers. Overall, one to two hours of dehydration was found to be optimum. In addition to the above, a reaction with a drying time of ~12 h was also performed over a total of 60 h. It was expected that such a long drying time might result in much higher breakdown, owing to possible thermal decomposition. Interestingly, the extent of breakdown was not very different in a 1 h dehydration time reaction *versus* a ~12 h dehydration time reaction, after accounting for the overall time differences. Higher oligomers, which were not resolved by HPLC, seem to have increased over this period, as is indicated by pronounced trailing (Figure S2, supplementary data). This suggests that the reaction is robust even when carried out for prolonged time periods (over days).

### 3.5. Optimum Reactant Ratios for Polymerization

**Figure 6:**
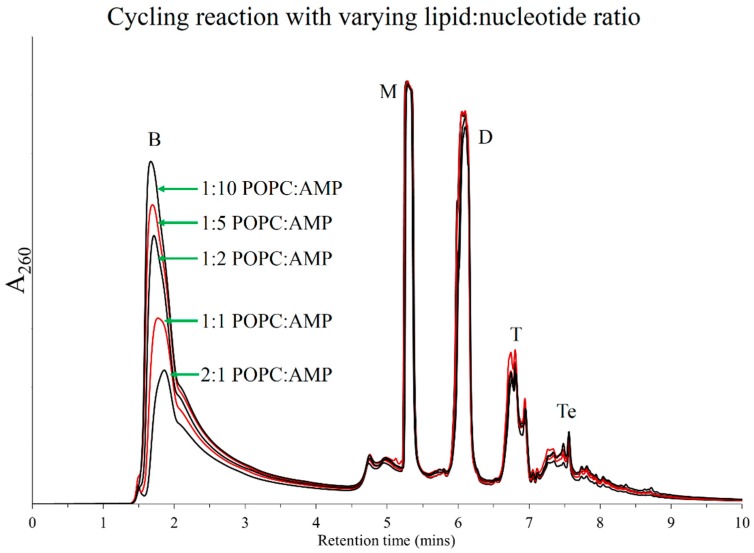
HPLC chromatograms obtained after seven DH-RH cycles using 5'-AMP (5 mM) and varying concentrations of POPC such that the lipid to nucleotide ratios decreased from 2:1 to 1:1, 1:2, 1:5, and finally 1:10. Higher lipid to nucleotide ratios resulted in reduced breakdown. All reactions were performed at pH 2.

On dehydration lipids form multi-lamellar sandwiches, which trap and arrange monomers within bilayers, resulting in concentration of monomers such that their reactive groups are potentially in close proximity [14]. However, the ratio of lipid to monomer will determine how the monomer arrangement is actually facilitated within each bilayer. For example, excess lipid will result in fewer monomer molecules being trapped per bilayer, thus limiting polymerization, whereas a very small ratio of lipid to monomer will also not confer the favorable organizational effect expected from lipids. Therefore, an optimum ratio of lipid to monomers might facilitate greater polymerization. To test this hypothesis, reaction mixtures with varying ratios of POPC: 5'-AMP were prepared. These were subjected to seven cycles of DH-RH at pH 2 using H_2_SO_4_ at 90 °C. As seen in Figure 6, a higher lipid to nucleotide ratio actually resulted in reduced breakdown, whereas the resultant yields of shorter oligomers varied only to a small degree.

### 3.6. Mass Analysis of Oligomers

**Figure 7:**
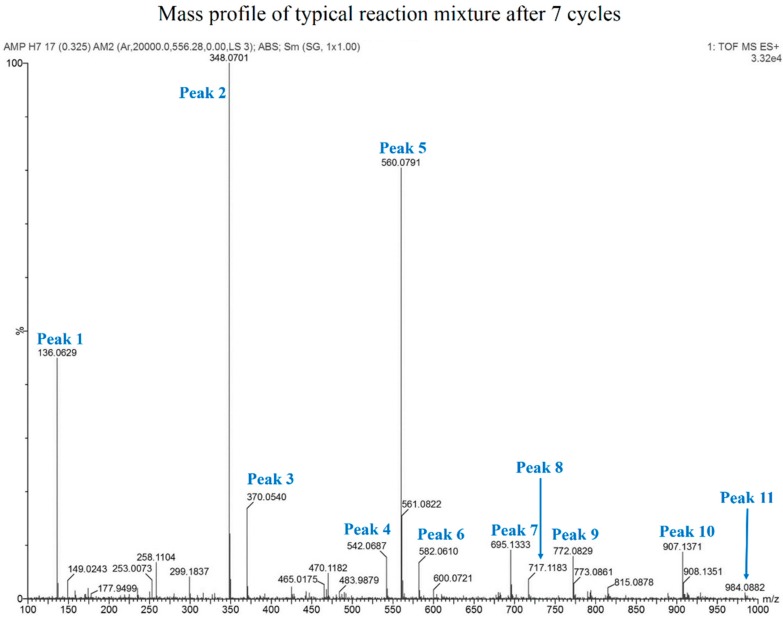
Mass profile of HRMS analysis obtained for Cyc7 sample of a typical reaction mixture of 5'-AMP and POPC. Chemical identities of the various peaks are tabulated in Table 1.

Since HPLC analysis demonstrated formation of oligomers during the DH-RH reaction in sufficient yields, mass analysis was performed to further characterize these oligomers. Aqueous phase from Cyc7 reaction mixtures, containing 5 mM 5'-AMP + 1 mM POPC, were used for mass determination. MALDI was initially performed on these samples. However, the material did not get ionized as expected. Possible reasons could be the presence of salt in the mixtures, as the starting material used was a sodium salt of 5'-AMP. Attempts to use C_18_ Zip-Tips (EMD Millipore) for reducing the salt content were not effective as it also resulted in loss of shorter oligomers in the process. Therefore, HRMS was employed for mass analysis of aqueous phases from the aforementioned mixture. Though the liquid chromatography element failed to resolve individual oligomeric peaks, it did eliminate the salt, resulting in mass profiles of whole reaction mixtures, as shown in Figure 7. Table 1 shows the theoretically calculated mass for the various oligomeric species formed in the reaction along with the actual observed masses obtained from HRMS analysis.

**Table 1:** Assignment of chemical identities to peaks from HRMS profile based on expected and observed mass numbers. Oligomers corresponding up to a tetramer were observed, albeit with abasic sites.

Peak No.	Assigned Species	Expected Mass	Observed Mass
1	Adenine	136.0617	136.0629
2	AMP	348.0703	348.0701
3	AMP + Na	370.0523	370.0540
4	Cyclic AMP Dimer minus (−) Base	542.0683	542.0687
5	AMP Dimer − Base	560.0789	560.0791
6	AMP Dimer − Base + Na	582.0608	582.0610
7	(AMP)2	695.1334	695.1333
8	(AMP)2 + Na	717.1153	717.1183
9	AMP Trimer − 2 Bases	772.0875	772.0829
10	(AMP)2 + Dimer − base	907.1420	907.1371
11	Tetramer − 3 Bases	984.0961	984.0882

From the masses observed, it was evident that the breakdown peak obtained during HPLC analysis was that of adenine and not adenosine. Importantly, mass for intact dimer was never observed in our various HRMS attempts. Instead, the observed numbers corresponded to a singly depurinated dimer. In one of the attempts, HRMS analysis was also performed on a purified dimer peak obtained from a typical reaction. The peak was collected from HPLC and lyophilized prior to subjecting it to HRMS analysis. The observed mass of this dimer peak affirmed the presence of a singly depurinated dimer. Similarly, masses obtained for higher oligomers (trimer and above) also showed that oligomers had abasic sites. Loss of base due to breaking of the N-glycosidic bond is known to take place at an enhanced rate at low pH and high temperatures [19,20]. Our results clearly indicate that the conditions that lead to increased polymerization also seem to promote loss of the informational moiety in the nascent oligomers.

### 3.7. Reaction with Other Nucleoside 5'-Monophosphates (5'-NMPs)

Purine nucleotides seem susceptible to loss of base at low pH and high temperature. To check if loss of base would take place in the case of other nucleobases as well, DH-RH reactions were carried out with the remaining 5'-ribonucleotides, including 5'-GMP, 5'-UMP and 5'-CMP. Since the proposed mechanism is thought to involve protonation of the phosphate followed by a nucleophilic attack by 2' or 3'-OH on the sugar of a neighboring monomer, the mechanism underlying phosphodiester formation should be independent of the base present. Hence, the conditions optimized for polymerization of 5'-AMP should also be applicable to these other 5'-NMPs. Given this, cycling reactions were performed with lipids using 5'-GMP, 5'-UMP, and 5'-CMP as the nucleotide component, either individually, or by mixing the cognate Watson–Crick bases (mixture of AMP + UMP or GMP + CMP in 1:1 ratio), or with all four nucleotides mixed in equal proportions. Figures S3 and S4 (supplementary data) show the HPLC chromatograms obtained for the above experiments using a DNAPac PA200 column. Since the column shows minimum base specific interactions at operational pH, retention times for monomers and higher oligomers should be the same for all nucleotides. As seen in the figure, reactions carried out with the four nucleotides individually resulted in oligomer formation but loss of base was predominantly observed only in the case of the purines. In reactions where equimolar mixtures of monomers capable of base pairing were used or even in the reaction with all four nucleotides, loss of base on cycling was seen. This could result from the purine nucleotides, as reactions containing only pyrimidine nucleotides showed no obvious breakdown. Preliminary mass analysis of the resultant mixtures from these different reactions also indicated formation of oligomers with abasic sites with a single base intact. Loss of base, which is observed in all these reactions, may be primarily due to low pH and high temperatures, which happen to be the same factors that promote phosphodiester bond formation in our reactions.

### 3.8. Reaction with Ribose 5'-Monophosphate (5'-rMP) to Form Sugar-Phosphate Oligomers

**Figure 8:**
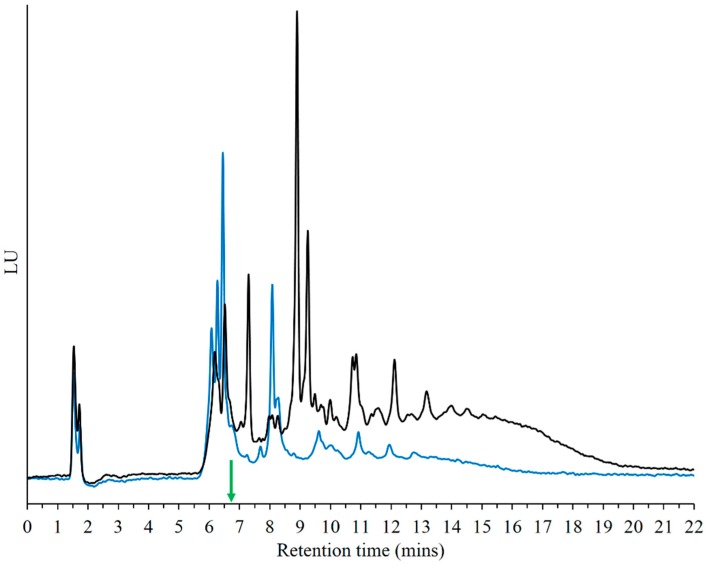
Comparison of Cyc1 and Cyc10 chromatograms from 5'-rMP reaction on DNA Pac PA200 column obtained using a fluorescence detector (λ_ex_ = 365 nm and λ_em_ = 425 nm). Polymerization takes place immediately after one cycle of DH-RH at pH 2, as is evident from the multiple peaks obtained (blue trace). Greater polymerization resulting in longer oligomers seems to occur when 5'-rMP monomers are subjected to 10 DH-RH; such cycles as indicated in the black trace. The green arrow denotes the retention time of 5'-AMP on the same gradient for comparison.

Since loss of base seems to be a predominant outcome under these reaction conditions, formation of oligomers with only sugar-phosphate backbones was investigated. Such sugar-phosphate polymers are interesting in themselves as they provide preformed polymeric templates that could be used to form informational polymers by addition of suitable bases. Given this interesting and prebiotically plausible scenario, 5'-rMP was subjected to DH-RH cycles under the aforementioned conditions, optimized for 5'-NMPs. Since the molecules lack a nucleobase, their detection or even the product analysis with spectrophotometric methods was challenging. However, weak intrinsic fluorescence was observed in samples, which potentially resulted from prolonged heating of sugars. It is important to note that the rMP monomer by itself does not show any such fluorescence. Using simultaneous excitation-emission analysis, the following wavelengths were shown to be the most relevant for detecting the rMP reaction products: λ_ex_ = 360–370 nm and λ_em_ = 420–430 nm. The reaction mixtures could now be analyzed with HPLC using a fluorescence detector. Multiple peaks were observed even in lower cycles, which resembled peaks that were obtained for nucleotide polymerization (Figure 8). Since assignment of individual peaks was not trivial, the HPLC peaks obtained from 5'-NMP polymerization were used as a proxy to temporarily assign oligomer length for peaks seen in the 5'-rMP reactions. As mentioned earlier, the rMP monomer alone could not be used as a control as it shows no fluorescence.

**Figure 9:**
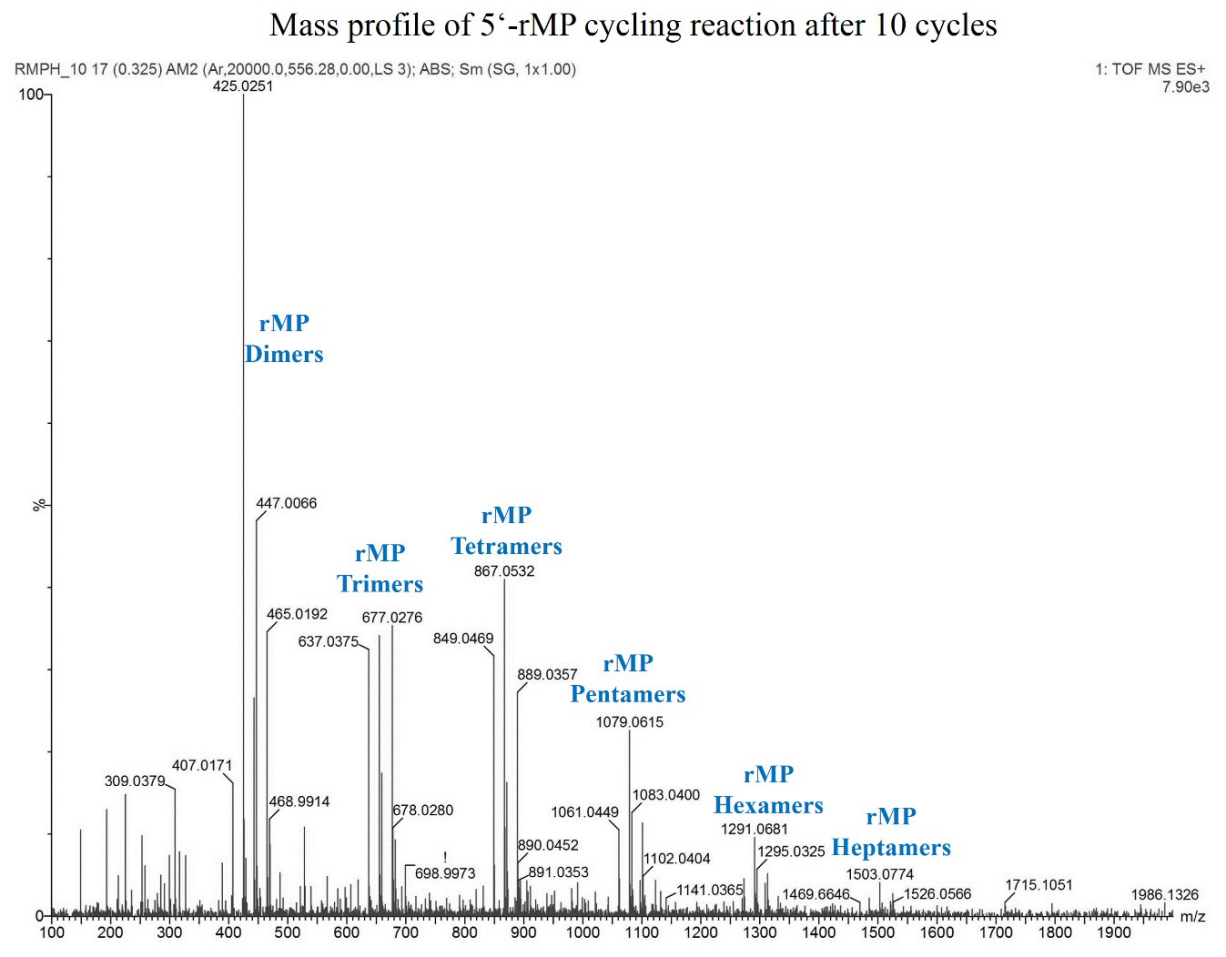
Mass profile of HRMS analysis obtained for Cyc10 sample of 5 mM rMP reaction, which was subjected to DH-RH cycles at pH 2 with 1 mM POPC. Observed mass numbers suggest that both cyclic and linear oligomers of rMP are formed, resulting in a cluster of mass peaks for each oligomer. Chemical identities of the various peaks are tabulated in Table 2. Heptamers may also be discerned but with less confidence due to reduced abundance.

The fact that even one DH-RH cycle resulted in several oligomer peaks (Figure 8) indicated that the kinetics for phosphodiester formation are probably very different in the case of 5'-NMP reactions *versus* rMP reactions. Formation of much higher oligomers was observed in chromatograms obtained from higher cycles, as indicated in Figure 8. Figure 9 shows the mass spectrum for an rMP-based reaction mixture obtained after 10 cycles of DH-RH at pH2. The observed mass numbers suggest that both cyclic and linear oligomers of rMP are formed in these reactions. Strand cyclization is known to occur as a result of intramolecular condensation, which is observed frequently during polymerization [21]. Formation of these sugar-phosphate backbones demonstrates the possibility of easily forming such oligomers under the low pH and high temperature conditions of volcanic geothermal pools. The exact chemical nature of these sugar-phosphate polymers is currently under investigation.

**Table 2:** Assignment of chemical identities to peaks up to hexamers from HRMS profile of rMP containing DH-RH reaction based on expected and observed mass numbers. Sodium adducts of following peaks were also obtained.

Chemical Species	Expected Mass	Observed Mass
Cyclic rMP Dimer	425.0244	425.0251
Linear rMP Dimer	443.0350	443.0363
Cyclic rMP Trimer	637.0330	637.0375
Linear rMP Trimer	655.0436	655.0424
Cyclic rMP Tetramer	849.0416	849.0469
Linear rMP Tetramer	867.0522	867.0532
Cyclic rMP Pentamer	1061.0502	1061.0449
Linear rMP Pentamer	1079.0608	1079.0615
Cyclic rMP Hexamer	1273.0588	1273.0591
Linear rMP Hexamer	1291.0694	1291.0681
Linear rMP Heptamer	1503.0780	1503.0774

## 4. Discussion

Lipid-assisted nonenzymatic polymerization of nucleoside 5'-monophosphates was observed under acidic conditions when DH-RH cycles were carried out at high temperatures. Several parameters that affect this reaction were varied to determine the optimal conditions that resulted in a good yield of oligomers, to enable further chemical characterization. In addition, varying the different parameters also lead to a better understanding of the environmental regimes that would have promoted similar polymerization reactions on prebiotic Earth. One of the first parameters we evaluated was pH. As mentioned earlier, the reaction is hypothesized to have a mechanism similar to that of acid-catalyzed esterification of carboxylic acids [15,16]. This means low pH should drive this reaction, which might enable the protonation of the phosphate group and facilitate a nucleophilic attack by the neighboring sugar’s 2'/3'-OH group, resulting in a phosphodiester bond. Our results corroborated that efficient polymerization was observed only when the reaction was carried out at a pH of 2 or below. Chemical polymerization of non-activated nucleotides on early Earth is, consequently, likely to have been facilitated in niches which were at low pH, e.g., acidic geothermal pools. The rehydrating agent used in these DH-RH cycles, therefore, should be able to maintain a low pH throughout the cycling period in order to promote good polymerization. Our characterization of this parameter using various acids showed that thermolabile acids or acids with a lower boiling point did not facilitate polymerization. Sulfuric acid promoted polymerization to the greatest extent in our study, followed by orthophosphoric acid. Incidentally, sulfuric acid is found in abundance in present-day volcanic geothermal pools. It is hypothesized to have also been present in similar niches on prebiotic Earth, which would have resulted from recurrent volcanic activity. The large amount of sulfur dioxide that would have constantly been released into the atmosphere might have resulted in the formation of acids similar to sulfuric acid (for example, sulfurous acid).

Loss of water from reaction environments is vital to condensation reactions. Additionally, the reduced water activity is favorable as it also reduces reverse hydrolysis of the growing oligomers, effectively increasing their yields. This can be facilitated efficiently by varying the temperature at which these reactions occur. We had hypothesized that higher temperatures would fare better. As postulated, reactions carried out at 90 °C and 105 °C yielded higher amounts of oligomers; however, 105 °C also resulted in increased breakdown products. Lower temperatures (60–75 °C) resulted in lesser breakdown but only yielded some oligomers. Higher polymerization may be achieved at lower temperatures by increasing the total number of DH-RH cycles that are carried out. Consequently, the optimum temperature for polymerization was found to be around 90 °C.

Removal of water can also be promoted by dehydration. However, rehydration phases of the DH-RH cycles are also important for lipid-assisted polymerization reactions, as has been explained earlier in Section 3.4. Importantly, rehydration phases allow the formation of vesicles from dried lipid lamellae, which enable encapsulation of the growing oligomers, protecting them from getting hydrolyzed. This potentially can also increase the yields of higher oligomers accrued over several cycles. Scenarios like this are thought to have set the stage for early forms of vesicular encapsulation of the genetic material, leading to the emergence of primitive cells. Our results indicated that a drying time of 1–2 h seemed optimum for good polymerization under laboratory time scales. Prolonged dehydration, representative of prebiotic environment time scales, also resulted in polymers, which persisted despite the greater drying time. This is indicative of the robustness of these polymerization reactions, which are shown to be favored at low pH and high temperatures, similar to what is thought to have been prevalent on early Earth.

Dehydrated lipid matrices drive these polymerization reactions by concentrating reactants in configurations that facilitate oligomer formation. Arrangement of monomers within the bilayers of these multi-lamellar organizing lipid matrices will hence depend upon the starting ratio of nucleotides and lipids in the reaction. At higher lipid to nucleotide (L:N) ratios, breakdown was considerably reduced but polymerization was not necessarily more efficient than in reactions that lacked lipids. On the other hand, at low L:N ratios, higher breakdown was observed and polymerization was only marginally improved. Our results suggest that L:N ratios of around 1:5–1:2 seem to be the optimum regime that furthered polymerization. This, however, cannot be directly extrapolated to origin of life scenarios as such fine control over reactant ratios was very unlikely. Importantly, complex amphiphiles such as phospholipids, which are prevalent in contemporary cells, were likely to have been represented by simpler amphiphilic molecules such as fatty acids on prebiotic Earth. In addition, amphiphilic structures formed by short hydrophobic peptides [22] also present an alternate scenario that could have provided an organization effect similar to that of lipids. Towards this end, it is pertinent to mention that formation of short peptides has shown to be promoted under a few different prebiotically relevant chemical conditions [23,24,25]. Two studies in particular also showed that formation of peptides from activated amino acids is enhanced in the presence of lipids [26,27]. Nonetheless, such studies enable us to delineate the role of amphiphiles in nonenzymatic polymerization reactions, providing a glimpse of how the two basic components of a primitive cell (informational molecules and the encapsulating membrane) might have interacted very early on, driving selection pressures that shaped their subsequent emergence.

The oligomers formed in these lipid-assisted syntheses were also subjected to preliminary denaturing PAGE analysis after radiolabeling with ^32^P. Streaks were observed on the gel (Figure S5, supplementary data), similar to the ones seen in the previous study [13]. Our preliminary analysis showed some loss of oligomers over cycling, which may be due to breakdown of both the starting reactant and the growing oligomers with multiple DH-RH cycles. This was seen as browning of reaction mixtures when 10 or more DH-RH cycles were carried out. One important aspect that might underlie this observation was the difference in the rehydrating agent used in these cycling reactions; H_2_SO_4_ was used in our reactions, while HCl was used in the previous study. Nonetheless, successful labeling of oligomers by polynucleotide kinase, post alkaline phosphatase treatment, and their electrophoretic behavior were indicative of formation of RNA-like oligomers in our reactions. Mass characterization of these RNA-like oligomers by HRMS analysis, however, revealed that the resultant oligomers have abasic sites. These sites result from the breakdown of the N-glycosyl bond between the sugar and the nitrogenous base, with the latter eluting as a breakdown peak during HPLC analysis. Loss of base is observed in short oligomers, which is indicated by their masses (Figure 7). A similar trend is expected to be observed even in the longer polymers, which we detected in our preliminary ^32^P analysis. These were, however, in very low concentrations for efficient detection by HPLC and hence show up only as trails (Figure 1).

Our study indicates that the resultant oligomers from lipid-assisted oligomerization reactions lose their informational moiety (the base) over the course of the reaction. This is not surprising, as abasic sites are known to be generated at high rates at low pH and high temperatures [20]. Since loss of base is unavoidable under the aforementioned conditions, we investigated the polymerization potential of ribose 5'-monophosphate monomers, which lack the base, under identical conditions. At low pH and high temperatures, 5'-rMP seems to polymerize much more efficiently when compared to the 5'-NMPs. Much longer oligomers were detected in the rMP reaction, as was evident from both HPLC and mass analysis. Formation of polymers from 5'-rMP, demonstrated for the very first time in this study, presents an interesting scenario in which sugar-phosphate oligomers can form readily on early Earth. Formation of such “prebiotic phosphodiester polymers” is thought to have resulted in stable intermediates during molecular selection, which would have been driven by chemical stability [28]. Such backbones could have sampled a vast informational space for different moieties that might have been added on, resulting in informational molecules that are thought to have populated a pre-RNA world.

Our study provides an important proof-of-principle result that supports the viable theory that the nucleobases we see in modern biology actually resulted from fine-tuning of an evolutionary process that originally selected moieties for prebiotically relevant characteristics like photo-stability, N-glycosyl bond stability, *etc.* [7]. Prior to the origin of informational molecules similar to modern RNAs, different heterocycles might have played the role of information storage entities. In a pre-RNA world, these bases could have been the predominant informational moieties, which would have eventually sequentially evolved into the informational molecules as we know them today [29]. Our results strongly suggest that extant nucleotides that have A, U, G, or C as the nitrogenous base cannot form informational polymers in acidic geothermal pools. These acid-catalyzed reactions result in oligomers with abasic sites as a consequence of their N-glycosidic bonds being labile to hydrolysis under low pH conditions. This is further corroborated by studies undertaken in the past that have attempted to form nucleosides with canonical bases; the yields obtained in these reactions were low [30]. On the contrary, recent studies that used other heterocyclic compounds resembling modern bases have shown formation of nucleosides to occur in higher yields under more prebiotically relevant conditions [31]. Such sugar-phosphate backbones in conjunction with other molecules, like amino acids, heterocyclic bases, and small peptides that would have been present in the prebiotic soup, can result in more complex prebiotic polymers, which may have constituted a proto-biopolymer world. In conclusion, formation of informational molecules from preformed sugar-phosphate backbones provides a prebiotically relevant and realistic pathway leading to informational molecules resembling modern RNAs.

## Supplementary Materials

Supplementary materials can be accessed at: http://www.mdpi.com/2075-1729/5/1/65/s1.
