# Supplementary material for: Characterization of RNA-Like Oligomers from Lipid-Assisted Nonenzymatic Synthesis: Implications for Origin of Informational Molecules on Early Earth
Source: Life (Basel). 2015 Jan 5;5(1):65–84. doi: 10.3390/life5010065 (PMC4390841; doi:10.3390/life5010065)
Supplement: Supplementary file 1 [file life-05-00065-s001.pdf]

Supplementary Data

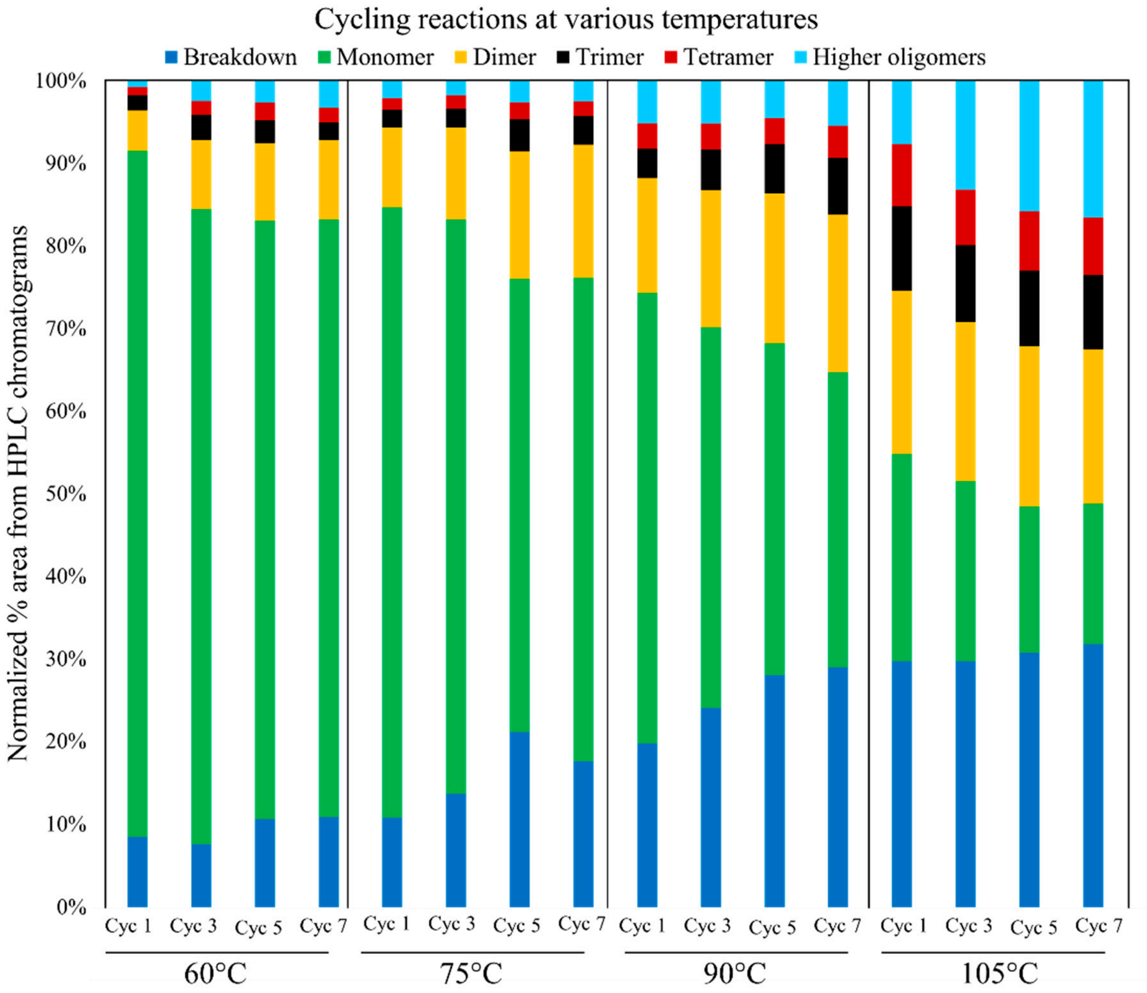

Figure S1. Comparison of polymerization reactions carried out at pH 2 over varying temperatures using graphical representation of chromatograms obtained for the different cycles. It is evident that lower temperatures result in lesser yields of oligomers as rate of back hydrolysis would be considerably higher due to more water activity. However, oligomers accumulated over cycles, as indicated by increased yields. At higher temperatures of 90 °C and 105 °C, polymerization did take place even in few cycles but breakdown was higher to begin with, especially in the latter. Trends in polymerization indicate that if more cycles at moderate temperatures are carried out they may result in yields comparable to that of reactions at higher temperatures with lesser cycles.

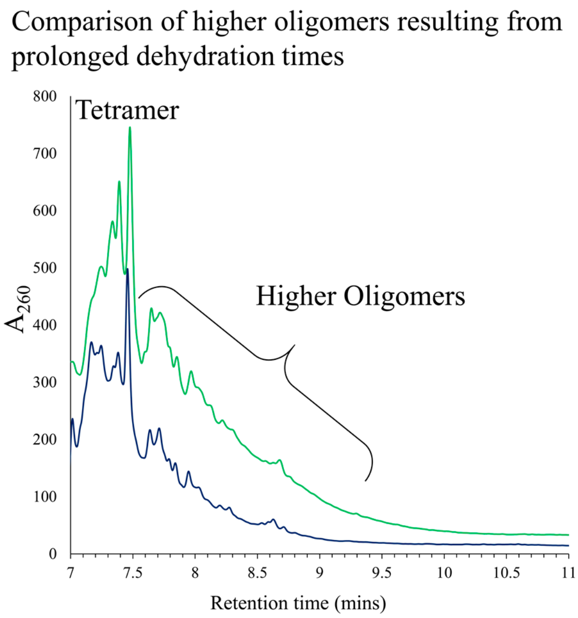

Figure S2. Chromatogram overlay showing increased tetramer yields and trailing in reaction with ~12 h dehydration time (green trace) when compared to a 1 h dry time reaction (blue trace). This alludes to the robustness of the reaction despite prolonged heating, which is more comparable to the length of a day-night cycle on Earth.

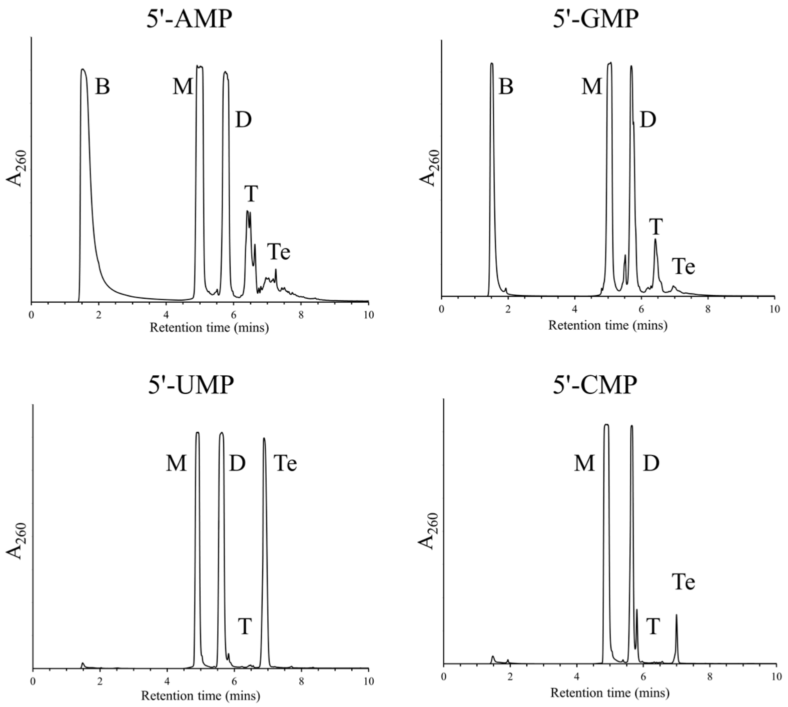

Figure S3. Cycling reactions were carried out using 5'-AMP, 5'-GMP, 5'-UMP and 5'-CMP, individually, to check for loss of base during the course of reaction. Below figure shows chromatograms from Cycle 5 of the cycling reactions carried out at 90 °C and pH 2 (using H_2SO_4). Peak identities for the pyrimidine reactions (bottom panels) were based on comparable elution times on DNAPac PA200 anion exchange column. As seen, breakdown peaks were observed only in the case of reactions involving purines.

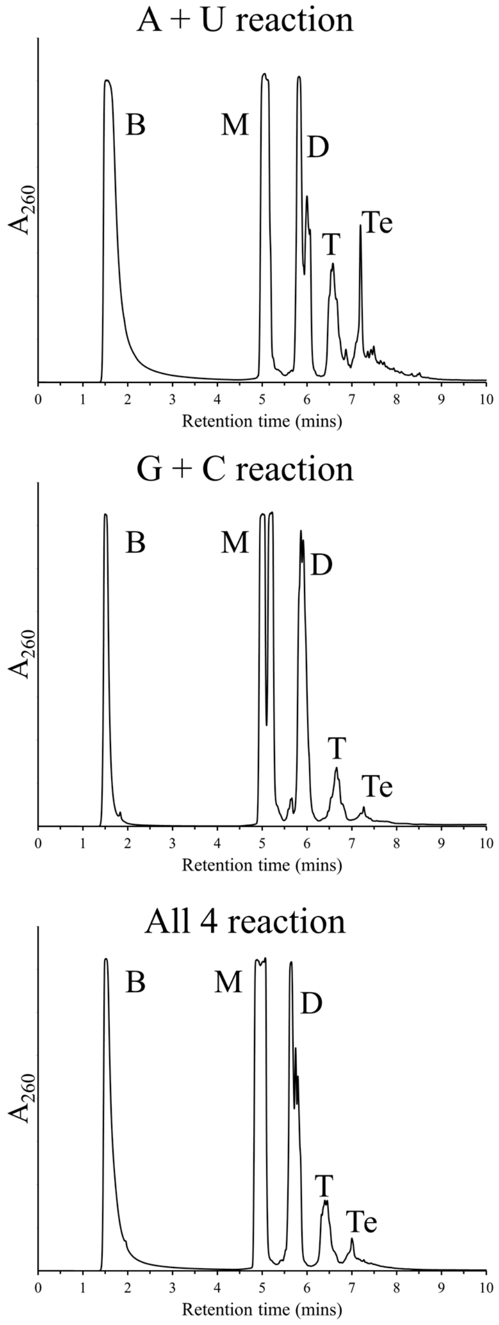

Figure S4. Cycling reactions were performed using monomers capable of base pairing (A+U and G+C in 1:1 proportions) and also with 1:1:1:1 ratio of all 4 nucleoside 5'-monophosphates under standardized reaction conditions. Chromatograms obtained for all cases showed breakdown peaks indicated in the below figure panels. This breakdown is thought to result predominantly from the loss of purines.

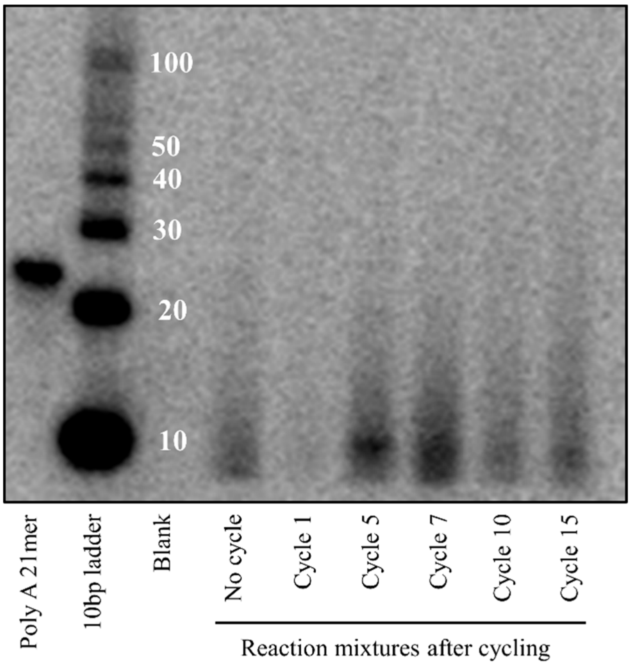

Figure S5. ^{32}P labelling of reaction products followed by preliminary PAGE analysis: Aqueous phase reaction mixtures, containing 27 mM 5'-AMP and 13 mM POPC, subjected to multiple cycles of DH-RH at 90 °C and pH 2 (using H_2SO_4), were used for radiolabelling. Samples were ethanol precipitated using sodium acetate and incubation at −20 °C overnight with 1 μL of GlycoBlue coprecipitant (Invitrogen—Life technologies). A fraction of these ethanol precipitated samples were treated with 1U of FastAP Thermosensitive Alkaline Phosphatase (Thermo scientific) under appropriate conditions to remove the 5'-PO_4. Excess enzyme was removed by phenol-chloroform-isoamyl extraction, followed by another round of ethanol precipitation. Pellets obtained were dissolved in 1X Polynucleotide Kinase (PNK) buffer and ^{32}P Labelling was performed using ^{32}P-γATP (1 μCi per sample) and 10U of T4 PNK (New England BioLabs Inc.) at 37 °C for 30 min. Excess unincorporated label was separated by spinning through Sephadex G-25 spin columns (GE Healthcare life sciences). Gel electrophoresis was performed on 20% denaturing (8 M urea) polyacrylamide gel (National Diagnostics). Controls used included 21mer Poly A (Thermo Scientific) and 10 bp ladder (Invitrogen) to estimate the size of the oligomers obtained in our reactions. Successful labelling of product oligomers, who’s mass indicates presence of abasic sites, with PNK, indicate the structural similarity that these oligomers share with nucleic acids. Also comparison with markers to estimate oligomer size distribution may not be appropriate as abasic oligomers will run differently on the gel.
